# Beyond the borders: The use of art participation for the promotion of health and well-being in Britain and Denmark

**DOI:** 10.1080/17533015.2013.817448

**Published:** 2013-07-11

**Authors:** Anita Jensen

**Affiliations:** GAIA Museum, Randers, Denmark

**Keywords:** visual arts, learning disabilities, mental health, social capital, well-being

## Abstract

**Background:**

This article compares British and Danish promotion of well-being through participation in art activity to empower the individual. It examines the influence of national, social and political contexts on art and health community projects by comparing practice and project outcomes.

**Method:**

Based on two case studies, the article draws on specific evidence in Britain and Denmark. The approach taken is one of the psychosocial inquiries allowing reflection on practice including participants’ testimonies.

**Results:**

The two cases showed comparable problems with restricted resources, funding and organisational limitations to service delivery. The British case study shows a bottom-up approach in contrast to the Danish case study where the approach is top-down. Although the benefits from participation in art activities in the two countries were influenced by a complex set of different interacting factors, outcomes were typically similarly positive: finding identity, feeling a sense of well-being and increased self-confidence.

**Conclusion:**

In terms of practice, policy and research and in the recognition of value of art participation, the comparison demonstrates how different stories, contexts and institutions engage in different ways to facilitate and enable service users as well as generating different challenges; recognising the benefits of developing best practice guidelines in art practice in health settings.

## Aims

### Objectives of This Article

The scope of arts and health community projects ranges from local to national or international, and includes music, visual arts, theatre, dance and literature. It reaches from the favelas in South America to the estates in Britain and the streets of Copenhagen and Hanoi. It includes residential homes for the elderly, mental health resource centres, hospitals and other venues across the world. This article examines the outcomes of art activity: does it have a positive impact on health and well-being – and if so, how? And how, considering issues related to bottom-up and top-down structural models, do the structures of arts and health organisations influence their service delivery? The article will also discuss the potential for developing guidelines, and considers if a sense of identity can be found through art participation.

### Well-Being

It is acknowledged that well-being is a problematic issue. Although it falls outside the scope of this article to provide an in-depth examination of the concept, a few observations are relevant here. Well-being might be seen as a ubiquitous term that occurs frequently and extensively in public and political discourse. But what is well-being? Is it subjective or objective? Is it a process or a state; and if it is a state, is it a neutral or a positive one? Or does it vary from individual to individual?

This article uses Foresight mental capital and well-being project’s (2008) definition of well-being “a dynamic state in which the individual is able to develop their potential, work productively and creatively, build strong and positive relationships with others and contribute to their community” (Foresight Mental Capital and Wellbeing Project, 2008, p. 10). The Foresight report goes on to note that well-being is enhanced when individuals are able to fulfill their potential and social goals, and to achieve a sense of purpose in society. Using the arts to increase well-being is widely recognised as an innovative healing approach with well-documented research demonstrating positive therapeutic outcomes for people with both physical and mental health problems (ACE, 2012; NEF, 2011; White, 2009; Wolf & Wolf, 2011). Research across an international arena suggests that engagement with the arts can have a significant positive impact, especially for people with a range of mental health problems (dementia, anxiety, depression, psychosis and substance misuse), and is widely used in healthcare settings in Britain, Ireland, Australia, Canada and the USA (ACI, 2010; White, 2009; Wikoff, 2004). Over the past decades, positive outcomes have supported further development in using the arts in the wider health sector.

### Social Capital

Features of social organisation, such as participation, reciprocity and trust in others, facilitate cooperation for mutual benefit (Williams, 1996) and can have a positive impact on health. In 2000, Putnam discovered that the most important factors in social connections were health and well-being, and that social capital is a well-established part of good health. This was contrary to his prior claims that health was not related to social capital (Putnam, 1995). According to Goleman (2005), emotional intelligence is connected with the ability to motivate oneself, and being productive and successful at what we do can reduce stress and increase stability. Although it is not typically referred to in such terms, social capital can be generated in community arts and health projects when successful conditions for improving social inclusion are achieved and/or there is an increased well-being of project participants. Art creation often inspires valuable dispositions including trust, openness, cooperativeness, tolerance and respect, and can be used as a tool to help creating social capital. Secker, Hacking, and Spandler (2008), in a review of participatory art projects for those with mental health problems, highlighted reduced social exclusion, improved mental health and, in particular, empowerment as key to generate social capital. These findings are supported by Spandler, Secker, Kent, Hacking, and Shenton (2008), in a study of various art and health initiatives, suggesting that art participation could be an important factor in recovery for mental health service users who live in a cycle of hopelessness and despair. The authors recommended that art and mental health provision should have an important role in the future of healthcare provision. Both the above articles’ conclusions recognise that approaches based on the social model of mental distress, underlined by empowerment and capacity building, can offer new ways of understanding mental health needs and promote recovery (Beresford, 2002). The experiences and expertise of the service user need to be valued (Duggan, Cooper, & Foster, 2002), and focus should be on the needs rather than on the classification, diagnosis or labelling (Milner & Kelly, 2009). Art participation delivered in a framework that empowers and provides focus on the needs of the service users has potential to become a catalyst for generating social capital.

### The British Context

In order to understand the context within which British arts and health organisations are operating, a more contextualised perspective is called for. Historically, the arts and community health organisations in Britain were often grass root initiatives (bottom-up) resulting from a perceived need to create alternative approaches to conventional medical models, in non-clinical settings. White (2009) identified a link between the arts and community health in Britain, and traced the origins of the arts and health initiatives to the late 1960s. He found that there were tensions from the start over whether the arts were to be seen as “an agent of social transformation or a mere instrumental tool” that could be used to meet government targets. In Britain, the benefits of participation in the arts was recognised, on a political level, by Alan Johnson, Secretary of State for Health (2008), when he described participatory art as “something that should be part of the mainstream – in both health and social care – and to provide best practice advice and streams of funding for art and health professionals.”^1^ Arts and health organisations were often driven by passionate individuals with a desire to make a difference and a belief in the positive therapeutic outcomes of art participation.

Although these organisations may have been seen as bearing some of the burden created by inadequacies in the public healthcare system, many eventually faced closure when funding was discontinued. With recent Art Council England funding cuts, the future has become uncertain for many.

### The Danish Context

Denmark does not have a tradition of grass root initiated arts and health initiatives, at least not to the extent of that in Britain. This may partly explain why, in Denmark, there are only a few projects that attempt to use creativity as a tool for well-being.

The apparent paucity of arts and health initiatives in Denmark could perhaps be due to the fact that the service users of the Danish healthcare system have not identified significant gaps in healthcare provision. Faced with new tendencies in society, including issues of inclusion and health inequalities (Dybbroe, Land, & Nielsen, 2012), there is potential for Denmark to find alternative ways to deal with these social issues. However, based on the current healthcare provision delivery in Denmark, potential initiatives are likely to be established with a top-down approach by local authorities, and less likely to be service user led or initiated.

We are presently witnessing some changes in other Nordic countries towards the inclusion of art and culture in tackling health-related issues. In Finland, in 2010, the Ministry of Education and Culture published an action programme aiming to promote health and well-being (and combat health inequalities) through culture and “to strengthen social inclusion at the individual, communal and societal level” (Liikanen, 2010, p. 8). In the action programme, the Finnish government recognised the need to take a cross-sector approach in future legislative reforms to promote health through culture. An overview of cultural activity and public health in Norway and Sweden concluded that the relationship between cultural participation and health promotion is growing and promising. Although Cuypers et al. (2011) found it difficult to draw conclusions at the research stage (the researched projects were ongoing), the overview indicated that none of the reviewed studies suggested negative effects. In Finland, Norway and Sweden, governments therefore see the potential of using arts and cultural activities to promote and improve health and well-being (Kulturpropositionen, 2009; Liikanen, 2010; SHD, KD og Norsk Kulturåd, 1999).

It remains to be seen if Denmark will make a significant effort to support mental health through a cultural policy. It is likely that with political recognition, the so far untapped potential of the role of art in health could play a significant role in future approaches to community and health initiatives in Denmark.

## Methods

This article compares two projects – one in Britain and one in Denmark – to explore how external factors can influence an art organisation’s ability to contribute to the well-being of its users. The two cases represent examples where art activity has been used to promote well-being, to create social capital and thereby to empower the individual. The cases are representatives for different approaches to arts and health community projects and thereby allow us to identify some of the strengths and weaknesses in a bottom-up versus a top-down structural approach.

The examination is based on evidence from each project. A psychosocial inquiry approach was used, which allowed reflection on practice while including participants’ testimonies. This approach also meant that the way that the external factors, such as policy and current agendas, influenced project delivery and outcomes could be considered. Although a comparison between two specific projects does not allow us to make any sweeping statements about the general approach of the two countries involved, it does allow us to identify pros and cons of the approaches applied in the two specific cases – even if the programmes varied greatly. This in turn may be useful for a future, more general discussion of the best way to use arts to promote health and well-being.

As specific examples, The Other Side Gallery (TOSG) in Britain and GAIA Academy in Denmark were selected. These two have important similarities:

- They worked in the context of community arts and health settings.
- They involved people with both physical disabilities and mental health issues.
- They were concerned with identity and well-being.

At the same time, the two cases differed as follows:

- They had different approaches to service delivery.
- They had different constitutions and funding structures.
- They were located in different parts of Europe.

The findings were based on case studies of TOSG and GAIA Academy. With regards to GAIA Academy, the examination was based on qualitative interviews that were conducted with three service users; sharing their experiences of being involved in the programme at GAIA Academy. The interviews were based on prior personal insight into the functioning of GAIA Academy. Moreover, both management and staff provided general information about the workings of GAIA Academy. With regards to TOSG, the examination was based upon testimonies from unpublished project evaluations covering the period from 2008 to 2012. The testimonies have been supplemented by email correspondence with one service user (service user, personal communication, 12 October 2012) and with general information provided by the management and staff of TOSG. Questions used for the interviews and in gathering data for the evaluation reports specifically covered issues around art participation and well-being relevant to the individual projects.

Service users from TOSG were males and females aged 18–76 years with mental health issues and some with physical disabilities. They were from diverse ethnic backgrounds and geographically based in Greater London, UK. Service users from GAIA Academy were males and females aged 16–24 years with physical disabilities (some profound) and mental health issues, from a Caucasian background, and who lived in the municipality of Randers in the North-West of Denmark. All service users gave consent to be quoted.

The author has worked with both organisations (and in other similar organisations). Particular efforts have been made to approach the topic with the purpose of relating to all issues without bias and to represent all views fairly. The primary interest of the author lies in presenting factual and neutral accounts of both organisations. The views and conclusions expressed are those of the author.

## Results

### The Other Side Gallery, Britain

Based on the premise that participation in art can increase well-being for the people with mental and other health issues, TOSG was constituted and registered as a charity in 2004. Founded in collaboration between service users and arts professionals, its aims have been to address the gaps in healthcare provision for people with various mental and physical disabilities, and to create opportunities for exhibition of artwork. TOSG operated with a service user involvement ethos and a group of service users also served as an advisory committee.

TOSG has worked with over 200 service user artists from various organisations and has exhibited over 2000 art works on its online gallery. It has been dedicated to the exhibition, promotion and sale of artwork from a community of people who generally have found it hard to interact with mainstream society due to various disabilities. The most considerable activity of TOSG has been the outreach programme, which enabled collaboration with other art initiatives in settings including hospitals, day care centres, old people’s homes and hostels for homeless people. TOSG also has supported service users to create work by facilitating art workshops when resources were available. Workshops and activities included printmaking, painting, portfolio preparation and national as well as international art partnerships. One such partnership was a collaboration with organisations in Europe. The service users visited the partners to meet other service users and to share art practice. Trips were also organised to support service users in gaining inspiration from other sources such as galleries and museums. Otherwise, individuals benefited from the art activities provided by their host organisation. To further the commitment towards supporting service user artists, in 2011, TOSG helped to create the Impact Arts Fair: the first international arts fair for marginalised artists held in Britain.

From inception, the main priority of TOSG has been to enable service users to exhibit their work in a professional manner in order to draw attention to the wealth of talent among disabled artists. The process of art participation has been described as both therapeutic and liberating. The feelings of engaging in therapeutic work were seen as a by-product of participating in art activity. A service user from TOSG has described this in the following terms:

A personal connection develops between the work and myself. I am not guessing the end product but travelling through an inner journey trying to make sense, a visual sense of the pictorial world. I am creating a different world to inhabit – a world of feeling/thoughts/identity. An alternative world maybe – sometimes of beauty.

Being able to exhibit works of art in established galleries was an empowering experience for many service users. In the words of other service users from TOSG (The Other Side Gallery, 2009):

I wasn’t totally confident that I could exhibit for the first time. Exhibiting work in public has been a huge hurdle for me. Now this has been overcome, I have been able to take a few more steps forward. I am currently putting some work up in my local library.

It has kick-started my creative side that I knew was there but has lain dormant for sadly too many years. I would be extremely pleased to be included in further exhibitions.

The positive impact of art participation is also illustrated by the testimony of another service user from TOSG (Rivers, 2008):

Art is not a panacea of all our difficulties or problems and it may not take away completely the problems we face on a daily basis – but it can make the quality of life that much better. It can help us engage with the creative process, have some fun, improve the quality of life and sense of well-being, gain new skills and interests and restore meaning by bringing people together through creativity.

These testimonies indicate that art participation had a positive impact on both well-being and self-confidence, and created a sense of achievement and recognition. One service user commented on an inner journey to create an alternative world – a place of beauty. Another service user spoke of how it had helped to find an old passion and a third suggested that art participation improved the quality of life. The testimonies further suggested that being an artist rather than a person with mental health problems or other disabilities could bring a new way of identifying with self. Practicing art could allow space for new connections to be made. Through art, individuals could possibly find opportunity to express feelings.

### GAIA Academy, Denmark

TOSG was partly created as a response to service users’ requests for exhibition opportunities, whereas GAIA Academy was created in response to a new government policy. In 2007, the Danish government set up an education programme directed at young people unable to access mainstream education. The programme was designed with a person-centred approach and aimed at the specific target group of 16–24 year with special educational needs. The purpose of the programme was to support individuals by offering skills to live an independent and active life and thus enhance chances for future employment.

The public GAIA Museum embraced this opportunity and set up the GAIA Academy. GAIA Museum already had a long history of working with disabled people as exhibitors of Outsider Art and as employees in sheltered workshops. When GAIA Museum opened its doors in 2002, it was partly with the vision of promoting inclusion by developing work processes within art and culture that allows active participation of disabled people.

GAIA Academy offered an arts and culture-focussed programme with the ethos that art participation increases the well-being of the whole person. The programme had a pedagogical approach with focus on well-being and social development, based on the assumption that art can be used as a means of expression. Positioned between arts participation and specialist education, it aimed to contribute to the development of young people with profound and complex learning issues and to support them on their journey to find a professional path and identity, and to support individuals’ move away from the label disabled, by increasing their self-confidence and supporting their artistic development. The spectrum of disabilities among service users included attention deficit hyperactivity disorder, mental health issues, physical disability and brain damage.

A project at GAIA Academy encouraged service users to work on the theme: *Who am I?* It provided an opportunity to examine identity and the prospect of looking at self with the identity of artist rather than disabled person. In an interview with the present author, service users stated that this project helped them finding an artist identity. A service user from GAIA Academy went on to say:

A person needs to be creative and I have proved that disability is not a barrier.

And another observed the following:

The project enabled me to look at my identity and to use some of my other skills. My art work included a mechanical function. I enjoy working with mechanics.

These testimonies made claims of developing skills and finding a positive identity. Thus for example, as reflected in the latter quotation, a service user realised the possibility of combining the satisfaction of working with mechanics in an art project and was therefore able to create a very personalised piece of work.

In sum, testimonies from both the British and the Danish cases suggested that both organisations encouraged service users to do more of what they do well and facilitated the concomitant benefits to well-being that accompanies fulfilment and achievement. Furthermore, the testimonies suggested that service users in both countries saw art participation as an essential part of their life; finding aspects of identity and self-confidence.

## Results

### GAIA Academy and TOSG

Although GAIA Academy and TOSG played similar roles in addressing mental health well-being through art activity, there were considerable differences between the two organisations. Service users attending GAIA Academy were considerably younger than those at TOSG and much additional time was spent communicating with parents or guardians. TOSG rarely had to involve guardians. According to Reynolds (2002), “art participation can enhance self and sensory awareness, stimulate thinking and encourage social skills, relationships and self esteem.” If we accept this view, we may ask whether art activity can be used in preventive health promotion targeting younger people? Is there an argument for early engagement of art participation with service users? Or can access to educational programmes such as GAIA Academy contribute to young service users’ well-being if more widely available?

### Identity

If we agree that art participation can support the well-being of an individual, then perhaps we can also consider a link between well-being and identity, noting that in their testimonies, service users from both organisations mentioned how art participation made a positive contribution towards a sense of identity. If a positive identity can indeed be formed through art participation, then society can partake in contributing to an individual’s identity – and it could be possible for people with mental health problems to find an answer to a social problem by redefining themselves as artists – and thereby restore their identity (Stickley, 2010). This was supported by Daykin, McClean, & Bunt (2007) who stated that art used as a therapy tool can be a means to reinstate identities that may have been damaged by the experience of illness. A crucial point for both TOSG and GAIA Academy was that service users were identified positively through their creative capacities rather than by a deficit model (e.g. ill, deficient or otherwise pathological). In contrast to traditional medical models of disability, TOSG and GAIA Academy advocated and promoted the cultural contribution their users had to offer. According to Samdahl (1992), a sense of empowerment and personal freedom may be felt through engagement in creative arts since “right” and “wrong” do not exist, creating less pressure to be “correct.” This contention is supported by Spandler et al. (2008), who recognised that art in health projects foster hope, creating a sense of meaning and purpose, including developing new coping mechanisms and rebuilding identities. In turn, this may also contribute to an acceptance of self and the voice of the artist rather than the disabled person. In this sense, both GAIA Academy and TOSG endeavoured to strengthen the individual’s understanding and acceptance of their own identity and abilities.

### Service Delivery/Funding

In terms of difference in service delivery, GAIA Academy provided a structured educational programme, whereas TOSG rarely delivered formal educational activity. Service delivery at GAIA Academy took place in accordance with the regulatory agreement with the local authorities, whereas TOSG’s services depended on the various terms and conditions established by the miscellaneous funding bodies supporting the organisation.

Despite the positive output from TOSG, it remains an organisation that could be described as oversubscribed, underfunded and surviving in a grey area between community activity and social service. TOSG rests on a mental health system without being fully independent and is often perceived as a mental health service rather than an independent art charity in its own right. It is reliant on funding from a variety of sources – not unlike other small charities – and consequently sustainability is a constant worry and the majority of resources are used on fundraising rather than on service delivery. In contrast, GAIA Academy (part of GAIA Museum) is partly funded by the local council. Even though the current economic climate in Denmark leaves the future of that source uncertain and subject to economic vulnerability, the fact that GAIA Museum (including GAIA Academy) is delivering healthcare provision on behalf of the local authorities means that it appears to stand in a much stronger economic position than TOSG. Both TOSG and GAIA Academy have national and international partnerships, which have brought developments in knowledge, stimulus and funding opportunities. However, limitations to resources have made it difficult for either organisation to fully utilise these partnerships.

### Creation of Social Capital

In terms of creating social capital, it could be argued that both organisations contributed to this process by creating “safe” environments to connect service users with common interests: participating in art projects. Requena (2003) had suggested that the importance of social capital lies in that it brings together several important sociological concepts, such as social support, integration and social cohesion. Both organisations enabled social activity extending beyond the art participation. For example, at GAIA Academy, the service users formed friendships outside the “classroom,” and TOSG involved service users in volunteering functions including hanging work and invigilating at exhibitions.

### Position

A notable difference between TOSG and GAIA Museum was the position in which each organisation existed. TOSG was recognised for its value in the art and health setting and was supported by healthcare professionals. In contrast, GAIA Academy had not yet gained recognition by health professionals on a wider scale. This lack of recognition of the various programmes at GAIA Museum by health professionals may change in the future, in particular, due to the collection of evidence of practice, the creation of more visibility in the cultural sector and the health sector.

### Bottom-Up and Top-Down

Another observation in relation to the background of the organisations is that TOSG was established by an apparent need vocalised by service users wanting to respond to a gap in healthcare provision. Service users were therefore active in the making of TOSG, which resulted in an important sense of ownership (bottom-up). In contrast, GAIA Academy was developed by GAIA Museum as a structured programme, resulting in less ownership for the service users (top-down). This may illustrate a general picture and reflect proactive service users at TOSG and more passive ones at GAIA Academy and, in turn, even be a reflection of some dissimilarity between the present situation in Britain and in Denmark in the arts and health field, arguably illustrating a difference between proactive and passive service users and models of engagement.

The bottom-up approach emphasises community participation and grass root movements. In contrast to the bottom-up approach, the top-down model is structured around the use of professional leadership (provided by external resources) that plans, implements and evaluates programmes (Macdonald, 1995). Such characteristics can be found at GAIA Academy. The bottom-up model creates partnerships with communities and professionals rather than leadership (Panda, 2007). This approach has been used by TOSG, attempting to create social sustainability. In general, it may seem that smaller grass root charities follow the bottom-up approach, whereas bigger and national organisations deploy top-down approaches in their activities – although at times the distinction between the bottom-up and top-down approaches becomes blurred.

If Denmark (or indeed any other country) is to employ models of engagement like those that have emerged in Britain, there are several lessons to be learned. As pointed out by Milner and Kelly (2009), attuning social practices within service settings to the requirements of disabled people is an obvious way to move from a disabling to an inclusive society. To create more inclusive models of practice, the argument could be made for a bottom-up approach, with the inclusion of service users to facilitate developments focussing on user-led solutions and involvement. The bottom-up approach is, however, often subject to financial vulnerability. The benefits of the top-down approach are that it might offer a stronger economic foundation if the initiatives were to be embedded in public authority health services, and that it may involve a more professional management – thereby being less dependent on limited resources. Both models have strengths and shortcomings and it is not a simple matter of one being obviously better suited to the arts and health than the other.

A possible – and important – disadvantage of delivery through healthcare services is that some people do not want to access these services due to stigma and other issues around diagnoses. This further perpetuates the benefits of a model of collaboration between the health and cultural sectors, which might help to identify a balance between the bottom-up (ownership, adoptability and outreach) and the top-down (financial security and professional leadership) approach, and to consider the benefits and disadvantages on a greater level.

### Creating False Hope

The findings also raised questions about the few cases where art participation created a negative impact. For example, an individual may create a false identity as an artist and have unrealistic expectations of what being an artist might mean. Although organisations need to be able to handle the ethical implications and dilemmas that arise when service users participate in art activity, they can often struggle to do so. Organisations may need to consider their limitations in order not to create a negative impact on the service users. For example, TOSG is not equipped to monitor and support individuals dealing with issues such as false identities and unachievable aspirations. Other criticisms of the work carried out at TOSG and GAIA Academy are based on the notion that the two organisations give a taste of hope to service users who are then, subsequently, faced by a lack of progression routes in the creative industry. With support and resources in tandem, it may be possible that some service users could maximise their participation in art activities, develop professional skills and further their involvement in the creative industry. But neither organisation is presently able to support a service user wanting to pursue an artistic career. This challenge is recognised and the gap in provision is acknowledged by both the organisations.

### Guidelines

To ensure best practice in the field, the Do No Harm principle (Primum Non Nocere) may be considered as part of an initial and on-going assessment of service users’ suitability for participation in art activity. It is a guiding principle for healthcare professionals to consider whenever an intervention or procedure is to be made and the patient’s well-being is the primary consideration. In the present context, the principle basically means that, given an existing problem, it may be better not to do anything than to do something and thereby risk causing more harm than good. It reminds healthcare providers that they must consider the possible harm that any intervention might produce. Implementing such assessment need not be complicated, but it does require a set of practical guidelines for the communication between artists working with service users and healthcare professional.

The need for a code of good practice guidelines has been identified in White’s (2010) paper “To develop a set of standards that reflect current best practice in the field; Greater awareness among those engaged in arts and health work and practical guidance and support for artists and healthcare professionals.”

White (2010) considered that good practice could provide a starting point to develop a benchmark tool for participatory arts-based approaches in healthcare settings. Such a tool could, among others things, function to improve standards, critical thinking and strategic planning. To be able to engage from a platform of sustainability, it is important that delivery does not take place in isolation, but takes place in partnership with local healthcare providers, social service and community organisations. To this end, in the view of the present author, the optimal application of the Do No Harm principle requires further exploration. With suitable adaptation, the principle has potential to be included in standardised good practice guidelines to the arts and health field. Furthermore, it could conceivably help to identify some limitations, and contribute to managing realistic expectations of the service users and limiting the creation of false hope, as well as creating confidence in arts and health practice.

## Conclusion

Both TOSG and GAIA Academy aspired to create social capital using participatory arts to promote health. In both examples, the well-being of service users improved through arts participation. In Denmark, policies promoting arts and health were generally poor or non-existing, whereas in Britain, although recognised as a useful tool in health promotion, it was nevertheless found that in both countries support and sustainability strategies for arts and health projects were significantly lacking. Denmark could, however, favourably look at research and models of engagement that have existed in Britain for decades.

Naturally, participating in community art is not the resolution to all mental or other health issues. However, it can be an important contributing factor to recovery and can act as a catalyst for creating social identity as well as becoming a platform for building self-confidence and improving social capital. Indeed, ultimately it can be a step towards greater integration into wider society.

In some parts of the world, community arts and health projects may have achieved much in terms of inclusion and tackling other social issues. However, without political support and recognition, it is virtually impossible to realise the potential. Political determination and engagement is needed in order to tackle issues associated with mental health and other disabilities.

## Footnotes

1 For the full text of Alan Johnson’s speech, see http://www.dh.gov.uk/en/News/Speeches/DH_088160
